# Comparative assessment on the probable mechanisms underlying the hepatorenal toxicity of commercial imidacloprid and hexaflumuron formulations in rats

**DOI:** 10.1007/s11356-021-18486-z

**Published:** 2022-01-07

**Authors:** Eman I. Hassanen, Ahmed M. Hussien, Sally Mehanna, Marwa A. Ibrahim, Neven H. Hassan

**Affiliations:** 1 Department of Pathology, Faculty of Veterinary Medicine, Cairo University, P.O. Box 12211, Giza, Egypt; 2 Department of Toxicology and Forensic Medicine, Faculty of Veterinary Medicine, Cairo University, Giza, Egypt; 3 Department of Animal Hygiene and Management, Faculty of Veterinary Medicine, Cairo University, Giza, Egypt; 4 Department of Biochemistry, Faculty of Veterinary Medicine, Cairo University, Giza, Egypt; 5 Department of Physiology, Faculty of Veterinary Medicine, Cairo University, Giza, Egypt

**Keywords:** Apoptosis, Hexaflumuron, Imidacloprid, Oxidative stress, Rats, Toxicity

## Abstract

Pesticides are viewed as a major wellspring of ecological contamination and causing serious risky consequences for people and animals. Imidacloprid (IM) and hexaflumuron (HFM) are extensively utilized insect poisons for crop assurance on the planet. A few investigations examined IM harmfulness in rodents, but its exact mechanism hasn’t been mentioned previously as well as the toxicity of HFM doesn’t elucidate yet. For this reason, the present study was designed to explore the mechanism of each IM and HFM–evoked rat liver and kidney toxicity and to understand its molecular mechanism. 21 male Wistar albino rats were divided into 3 groups, as follows: group (1), normal saline; group (2), IM; and group (3), HFM. Both insecticides were orally administered every day for 28 days at a dose equal to 1/10 LD50 from the active ingredient. After 28 days postdosing, rats were anesthetized to collect blood samples then euthanized to collect liver and kidney tissue specimens. The results showed marked changes in walking, body tension, alertness, and head movement with a significant reduction in rats’ body weight in both IM and HFM receiving groups. Significant increases in MDA levels and decrease of GHS levels were recorded in liver and kidney homogenates of either IM or HFM groups. Liver and kidney tissues obtained from both pesticide receiving groups showed extensive histopathological alterations with a significant increase in the serum levels of ALT, AST, urea, and creatinine and a decrease in total proteins, albumin, and globulin levels. In addition, there was upregulation of the transcript levels of casp-3, JNK, and HO-1 genes with strong immunopositivity of casp-3, TNF-ὰ, and NF-_K_B protein expressions in the liver and kidneys of rats receiving either IM or HFM compared with the control group. In all studied parameters, HFM caused hepatorenal toxicity more than those induced by IM. We can conclude that each IM and HFM provoked liver and kidneys damage through overproduction of ROS, activation of NF-_K_B signaling pathways and mitochondrial/JNK-dependent apoptosis pathway.

## Introduction

Exposure to ecological contamination stays a significant wellspring of safety hazards around the world, particularly in agricultural nations, where destitution, absence of interest in current innovation, and feeble natural enactment join to cause high contamination levels. Among the ecological contamination, overexposure to pesticides is viewed as one of the causative components of different diseases in humans and livestock (Cheng et al. 2011). Pesticides have discovered broad applications in horticultural and veterinary practices and have the potential for accidental effects on natural life, human and domesticated animals (De et al 2014). Humans and animals repeatedly presented to pesticides through water or food (Fisk 2007). Persistent pesticides delivered in one area of the world can be moved through the air to other areas through a continuous cycle of evaporation and deposition (Koirala et al. 2007).

Imidacloprid, 1[(6-chloro-3-pyridinyl) methyl]-N-nitro-2-imidazolidinimine, is a neonicotinoid insecticide widely used to fight pests of cereals, fruits, and vegetables due to its low soil persistence and high insecticidal activity at a low application rate (Casida and Durkin 2013). Imidacloprid (IM) can exaggerate the toxic properties and adverse effects which may be fatal for human and animal health (Benjamin et al. 2006; Lv et al. 2020). Several studies reported that IM is causing severe hepatotoxicity, nephrotoxicity, male infertility, and neurological disorders in mice (Bhardwaj et al. 2010; Li et al. 2021). Another example of broadly used insecticides is hexaflumuron (HFM), Benzoylphenyl urea (BPU) insecticide; it is an insect growth regulator that works by inhibiting a chitin synthesis (Khajepour et al. 2012). Its active ingredient is categorized as unlikely toxic to humans while one recent study about its local formulation showed hepatotoxicity and immunotoxicity in rats but with unclear mechanism of action (Noaishi et al. 2019).

Oxidative stress plays an important role in most pesticides-inducing toxicity that is leading to free-radicle related cell and DNA damage (AlBasher et al. 2020). Oxidative mechanisms have a pivotal role in insecticide-induced tissue damage not only by balancing oxidant-antioxidant status but also by inhibiting neutrophil infiltration and regulating inflammatory mediators (Delgado et al. 2006; Muniz et al. 2008). Another important mechanism of insecticide-induced cytotoxicity is the programmed cell death via the intrinsic and extrinsic pathway of apoptosis (Abdel-Daim and Abdeen 2018). The intrinsic pathway of apoptosis begins after cellular injury and is mediated by intrinsic oxidative stress and several factors as p53 and JNK that are activated by ROS (Peter 2011). P53 and JNK are the critical activators of the intrinsic pathway via stimulating the pro-apoptotic proteins (Bax) and suppressing the anti-apoptotic proteins (Bcl2) (Cheng and Chen 2010). The extrinsic apoptotic pathway is mediated via several death ligands and receptors present on the cell surface as Fac, TNFR1, Apo2 and Apo3 (Muntané 2011). All of these receptors induced apoptosis via activation of caspase cascade involving casp-8, casp-3, casp-9 either directly or indirectly by NF-_k_B signaling pathway that mediated by several cytokines as TNF-ὰ, IL-1 causing apoptosis and inflammatory reactions (Bradley 2008). Furthermore, it is reported that ROS increased the mitochondrial membrane permeability through opening the calcium transport channels leading to Cyt-C release and initiates the casp-3 dependent apoptosis (Abdel-Daim et al. 2019; Sayed et al. 2019).

By increasing the applications of IM and HFM in agricultural and veterinary practices, the hazards increased for consumers via intake of fruits and vegetables containing the pesticide remains. In addition, the possible mechanism of IM and HFM-induced toxicity in non-target organisms remains to be elucidated; although, it is important to understand the toxic mechanism of any commercial pesticides to minimizing the health risk for consumers. Hence, the current study aimed to investigate the possible mechanisms of IM and HFM–induced hepatorenal toxicity in rats with comprehensive insights on the role of the JNK/Bcl2 protein family and TNF/NF-_K_B signaling pathway in such toxicity.

## Materials and methods

### Chemicals

The study was conducted using the commercial formulations of pesticides obtained from Kafr El-Zayat Pesticides & Chemicals Company (Kafr El-Zayat, Gharbia, Egypt). Marketed hexaflumuron (Dimeuron® 10% EC, 1-[3,5-dichloro-4-(1,1,2,2-tetrafluoroethoxy) phenyl]-3-(2,6-difluorobenzoyl) urea with chemical formula C_16_H_8_Cl_2_F_6_N_2_O_3_), and marketed imidacloprid (Imidacloprid® 70% WP, (NE)-N-[1-[(6-chloropyridin-3-yl) methyl] imidazolidine-2-ylidene] nitroamide with chemical formula: C_9_H_10_ClN_5_O_2_) were freshly prepared in deionized distilled water according to the required concentration of the active ingredient.

### Animals and animal grouping

Twenty-one male albino Wistar rats (170 ± 20 g) were obtained from the Department of Veterinary Hygiene and Management’s Animal House, Faculty of Veterinary Medicine, Cairo University, Egypt. Animals were reared in plastic cages, fed with standard commercial pelleted feed, and water was supplied ad libitum. They were inspected for health status and acclimatized to the research laboratory environment for two weeks before use. All the procedures and experimental design were permitted by the institutional animal care and use committee (IACUC) of Cairo University (approval number: Vet CU12102021361).

Rats were randomly divided into 3 groups (n = 7) and were given the following materials every day by oral gavage for 28 days. Group (1) received normal saline and was kept as a control negative group. Group (2) received IM at 45 mg active ingredient/kg bwt representing 1/10 LD50 and equal 64.3 mg/kg bwt from the marketed IM 70%. Group (3) received HFM at 11 mg active ingredient/kg bwt representing 1/10 LD50 and equal 0.11 ml from the marketed HFM 10% that dissolved in 0.9 ml deionized distilled water. Doses of pesticides were selected based on their LD50 which was reported to be 450 mg/kg bwt for IM, and 110 mg/kg bwt for HFM formulations (Mikolić and Karačonji, 2018; Noaishi et al. 2019; WHO, 2020). The selected dose of both IM and HFM was equivalent to human dosage levels of 328- and 80 mg/kg bwt, respectively. Humans can be repeatedly exposed to these high dosage levels due to the high potential of both insecticides for bioaccumulation in the food chain relating to their high persistence in the environment and their low biodegradation (Bonmatin et al. 2005).

Rats in all groups were daily observed for any clinical signs and mortality as well as weighed weekly up to the end of the experimental period.

### Behavioral parameters

After 28 successful days, four representative behaviors include alteration in walking, body tension, alertness, and head movement were observed and evaluated as the markers of hepatorenal toxicity according to the method described by Hayase et al. (2000), but with some modifications. These behaviors were scored to determine the degree of behavioral changes and difference between groups and described on a graded scale as the following: 0 = normal behavior, 1 = light changes, 2 = mild changes, 3 = moderate changes, and 4 = severe changes.

### Sampling

After the behavioral assessments, rats were anesthetized with intramuscular injections of xylazine (10 mg/kg) and ketamine (90 mg/kg) to collect blood samples from the orbital sinus, then centrifuged at 3500 rpm for 5 min to obtain clear serum samples preserved at − 20 °C till used for biochemical analysis. After that, rats were euthanized by cervical dislocation to collect liver and kidney samples. Part of these samples was preserved at − 80 °C till used for oxidative stress evaluation and molecular studies while the other part was fixed in 10% neutral buffered formalin to perform histopathological and immunohistochemical examinations.

### Biochemical enzyme markers

Alanine aminotransferase (ALT), aspartate aminotransaminase (AST), total proteins, albumin, glucose, total cholesterol, high-density lipoproteins cholesterol (HDL-C), triglycerides (TG), urea, and creatinine were measured in the collected serum samples using standard kits Marketed by (SPECTRUM-Germany) according to the constructions of the manufacturer. Low-density lipoproteins cholesterol (LDL-C) concentration was calculated according to the method of Friedewald and Levy (1972). Globulin concentration was calculated from the difference between total proteins and albumin concentrations.

### Oxidative stress evaluations

The frozen liver and kidney tissue samples were homogenized using the cold buffer. Afterward, lipid peroxidation was determined by measuring the levels of MDA spectrophotometrically at 534 nm according to the method of Ohkawa et al. (1979). The antioxidant GSH content was measured at 405 nm following the method of Koracevic et al. (2001) by using the manufacturer kits (Biodiagnosic Com, Cairo, Egypt).

### Histopathological examinations 

Formalin-fixed liver and kidney tissue specimens were dehydrated using the ascending grade of ethanol, purified by Xylene, embedded in paraffin wax and sliced at 4.5 μm to obtain paraffin-embedded tissue sections stained by H&E and examined under light Olympus microscope to determine any pathological alterations (Bancroft 2012).

All the observable pathological parameters were graded using a classical semiquantitative scoring system to assess the degree of lesion severity between different groups. Five-pointed ordinal scale used as the following: (0) none, (1) mild < 25%, (2) moderate 25%:50%, (3) severe 50%:75%, and (4) extensive severe > 75% tissue damage (Hassanen et al. 2021).

### Immunohistochemical studies

Immunohistochemical examination was performed to determine the protein expression of casp-3 (a marker for apoptosis), Nuclear factor-_K_ protein (NF-_K_B) and tumor necrosis factor alfa (TNF-ὰ) as markers for inflammation in liver and kidney tissue sections using avidin–biotin-peroxidase complex (ABC). Briefly, deparaffinized tissue sections were incubated with different primary antibodies (Abcam Ltd., USA) then incubated with the reagents required for ABC reaction (Vectastain ABC-HRP Kit, Vector Laboratories). Afterward, slides were labeled with peroxidase and colored with DAB-chromogen substrate (Sigma), then examined under a light Olympus microscope.

The mean percentage area of various immunostaining expressions in different groups was determined by using Image J software.

### Quantitative RT-PCR for casp-3, JNK, HO-1, and Keap-1 genes

The total RNA was extracted using the RNeasy Mini Kit (Qiagen Cat No./ID: 74,104) according to the instructions provided. The synthesis of the first-strand cDNA was performed using SuperScript Reverse Transcriptase (Thermo Scientific) according to the manufacturer’s instructions. Quantitative real-time PCR was done using SYBR™ Green PCR Master Mix (Thermo scientific Cat number: 4309155) by the ABI Prism Step One Plus Real-Time PCR System (Applied Biosystems). The assay was performed in duplicates and the ACTB was used as the internal standard for the calculation of the expression level. The primer sets of the studied genes were shown in Table 1 and the fold change was calculated using 2^−˄˄CT^.

**Table 1:** The primer sets of the studied genes

	Sense	Antisense	Amplicon	Accession no
Caspase 3	GAGCTTGGAACGCGAAGAAA	TTGCGAGCTGACATTCCAGT	221	NM_012922.2
JNK	GTCATTCTCGGCATGGGCTA	TGGACGCATCTATCACCAGC	337	NM_053829.2
HO-1	AGCGAAACAAGCAGAACCCA	ACCTCGTGGAGACGCTTTAC	166	NM_012580.2
Keap-1	ATGTGATGAACGGGGCAGTC	AAGAACTCCTCCTCCCCGAA	190	NM_057152.2
***ACTB***	CCGCGAGTACAACCTTCTTG	CAGTTGGTGACAATGCCGTG	297	NM_031144.3

*JNK*, c-Jun N-terminal kinases; *HO-1*, Heme oxygenase; *Keap-1*, Kelch Like ECH Associated Protein 1; *ACTB*, Beta actin (housekeeping gene)

### Statistical analysis

All the parametric values were illustrated as means ± standard deviation of the mean (SD). The recorded results were examined by one-way analysis of variance (ANOVA) and post hoc Duncan’s test using the statistical package program (SPSS version 25); P values < 0.05 represent statistical significance. Nonparametric values as the behavioral assessments and histopathological scoring were expressed as median and analyzed by using Kruskal–Wallis H test followed by the Mann–Whitney *U* test.

## Results

### Clinical signs and body weight of rats

Rats in different groups didn’t show specific clinical signs and mortality all over the experimental period. Regarding body weight, administration of either imidacloprid or hexaflumuron caused a significant decrease in the average body weight of rats compared to the control group. Furthermore, hexaflumuron showed a significant decrease in body weight compared to imidacloprid exposed group (Table 2).

**Table 2:** Effects of hexaflumuron and imidacloprid on body weight (g) of treated rats

Group/weeks	Control	Imidacloprid	Hexaflumuron
W1	158.2 ± 3 ^a^	158.8 ± 3.5 ^a^	149.4 ± 2.5 ^a^
W2	177.6 ± 2.8 ^a^	155.6 ± 2.5 ^ac^	146.8 ± 3 ^bc^
W3	198 ± 5 ^a^	153.6 ± 2.3 ^ac^	153.6 ± 2.3 ^bc^
W4	219.4 ± 5 ^a^	148.2 ± 3.3 ^ac^	148.2 ± 3 ^bc^

Values are presented as mean ± SD. (*n* = 5 rat/group). Values with different letters are significantly different at *P* ≤ 0.05

### Behavioral observations

Rats receiving either IM or HFM showed severe abnormalities in walking, body tension, drowsiness, and head movement that were significantly different from those of the control group. Furthermore, there was a significant increase in the aforementioned symptoms observed in HFM receiving group compared with those of imidacloprid treated rats (Table 3).

**Table 3:** Effects of imidacloprid and hexaflumuron on some behavioral changes in rats

Group/parameters	Control	Imidacloprid	Hexaflumuron
Walking	0^a^	3^b^	4^c^
Body tension	0^a^	3^b^	4^c^
Alertness	0^a^	4^b^	4^b^
Head movement	0^a^	3^b^	3^b^

Data expressed as Median. (*N* = 5 rats/group), values having different letters in the same row means significantly different at *p* ≤ 0.05

0, normal behavioral patterns; 3, moderate behavioral alterations; 4, severe behavioral alterations

### Biochemical enzyme markers

Data presented in Table 4 showed that administration of both IM and HFM in rats led to a significant elevation in the entire liver biomarkers if compared with the control group. Moreover, HFM exposure showed a significant increase in serum AST and ALT activities in comparison with the imidacloprid group. On the other hand, total proteins, albumin and globulin levels were significantly inhibited in IM and HFM groups when compared to the control group. While there were no significant differences in total proteins, albumin and globulin levels between imidacloprid and hexaflumuron groups.

**Table 4:** The effect of imidacloprid and hexaflumuron on serum biochemical parameters in rats

Hexaflumuron	Imidacloprid	Control	Group/parameters
70.88 ± 4.98 ^c^	63.02 ± 4.36 ^b^	40.89 ± 2.72 ^a^	ALT (U/L)
102.70 ± 4.77 ^c^	79.52 ± 5.91 ^b^	57.66 ± 4.69 ^a^	AST (U/L)
5.82 ± 0.94^b^	6.50 ± 0.90 ^b^	8.60 ± 0.52 ^a^	TP (g/dl)
3.43 ± 0.34 ^b^	3.56 ± 0.62 ^b^	4.52 ± 0.36 ^a^	Albumin (g/dl)
3.18 ± 1.01^b^	3.01 ± 0.37 ^b^	4.14 ± 0.38 ^a^	Globulin (g/dl)
180.74 ± 5.63 ^c^	122 ± 5.43 ^b^	83.80 ± 7.19 ^a^	Glucose (mg/dl)
137.96 ± 9.23 ^c^	116.10 ± 7.13 ^b^	74.43 ± 7.74 ^a^	Total cholesterol (mg/dl)
17.78 ± 9.18 ^c^	31.96 ± 4.55 ^b^	41.25 ± 5.21 ^a^	HDL-C (mg/dl)
103.27 ± 8.00 ^c^	68.43 ± 10.4 ^b^	23.86 ± 5.00 ^a^	LDL-C (mg/dl)
84.83 ± 7.42 ^b^	78.35 ± 6.72 ^b^	46.16 ± 4.40 ^a^	Triglycerides (mg/dl)
61.04 ± 3.53 ^c^	45.76 ± 3.29 ^b^	33.52 ± 3.48 ^a^	Urea (mg/dl)
1.97 ± 0.17 c	1.40 ± 0.18 b	0.78 ± 0.25 a	Creatinine (mg/dl)

Data expressed as Mean ± SD. (*N* = 5/group), a; b; c means having different superscript letters in the same row differ significantly at *p* ≤ 0.05

Abbreviations: ***AST***, aspartate amino transferase; ***ALT***, alanine aminotransferase; ***TP***, total proteins; ***HDL-C***, high density lipoprotein cholesterol; and ***LDL-C***, low density lipoprotein cholesterol

Concerning kidney function parameters, there was a significant increase in serum urea and creatinine levels in groups receiving either IM or HFM if compared with the control group, but the HFM receiving group showed the highest levels.

In the case of glucose and lipid profile, there was a significant elevation in serum glucose levels in both IM and HFM exposed groups in comparison with the control group but, the highest levels were observed in the hexaflumuron group. Serum total cholesterol, TG and LDL concentrations were significantly elevated in IM and HFM groups in comparison with the control group. The highest total cholesterol and LDL levels were observed in the HFM group, while triglycerides concentration was the same in both treated groups. Regarding serum HDL concentration, there was a significant decline in both IM and HFM exposed groups when compared to the control group, but the lowest levels were detected in the HFM group.

### Oxidative stress evaluations

**Fig. 1:**
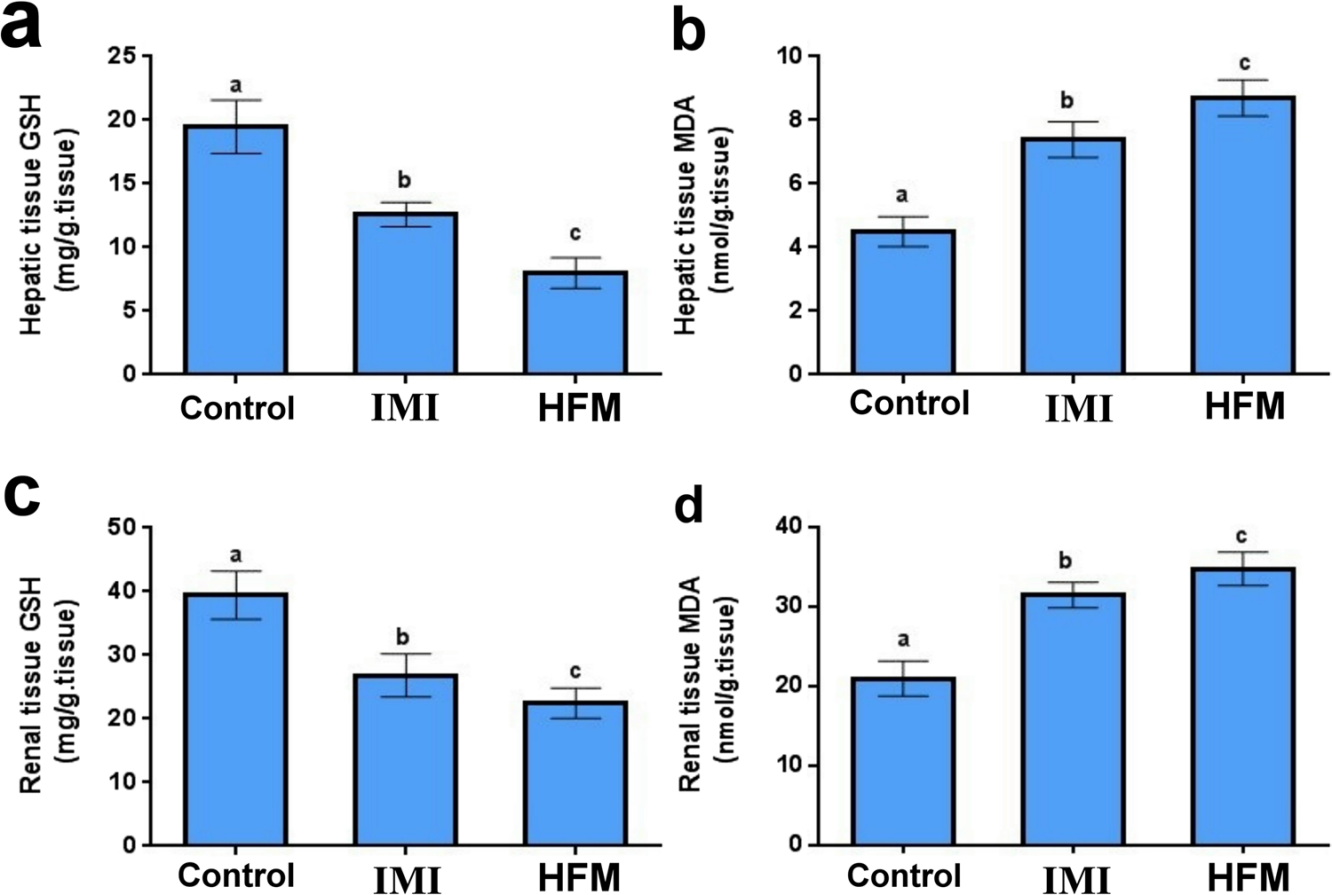
Effects of imidacloprid and hexaflumuron on hepatic and renal MDA level and GSH content of male rats. Values are presented as mean ± SD. (n = 5 rat/group). Values with different letters are significantly different at P ≤ 0.05. Control (C), Imidacloprid (IM) and hexaflumuron (HFM), Malondialdehyde (MDA), Reduced Glutathione (GSH)

The highest MDA levels and lowest GSH levels were observed in the group receiving HFM. Furthermore, IM receiving group showed a significant elevation in MDA levels and a decrease in GSH levels compared with the control group (Fig. 1).

### Histopathological examinations

**Fig. 2:**
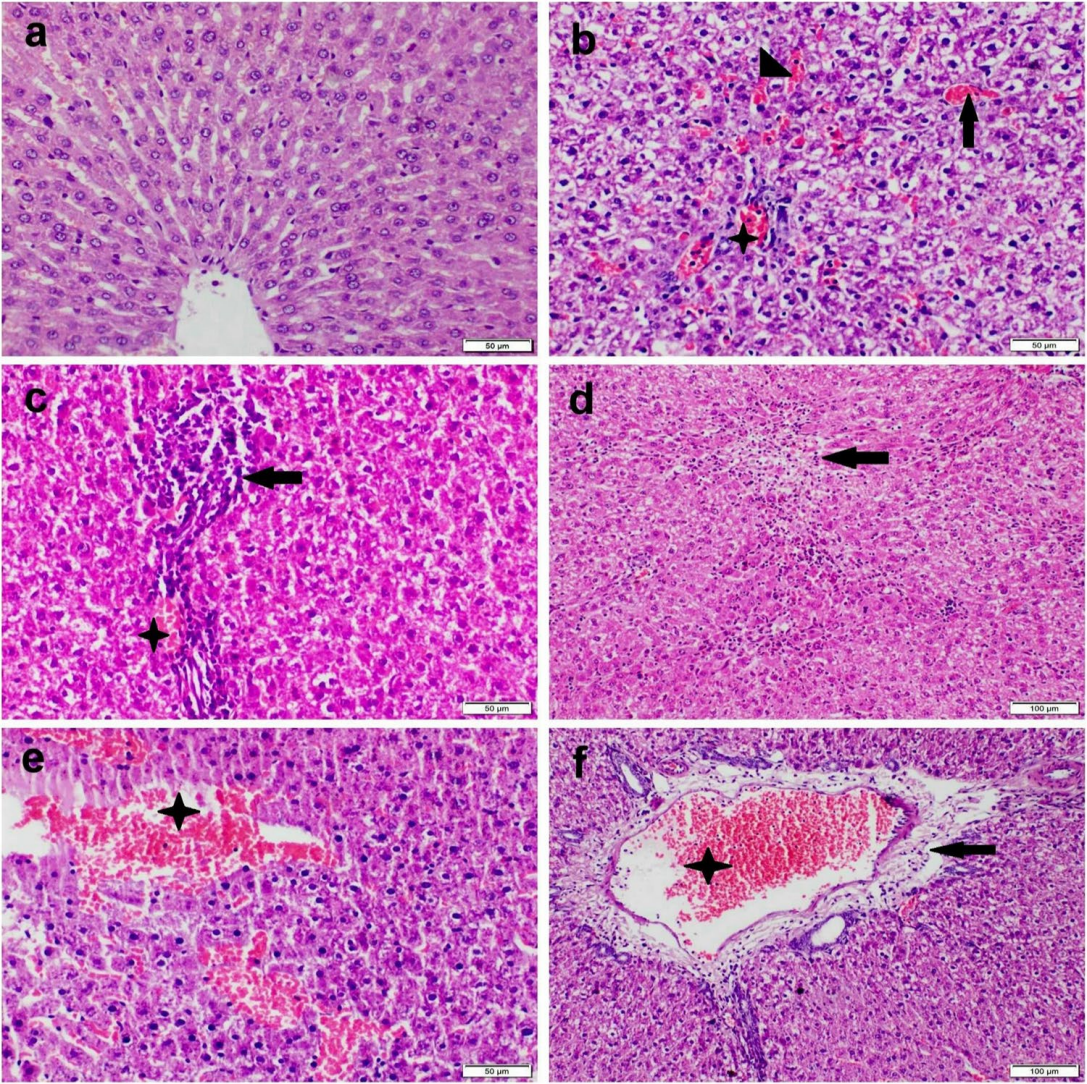
Photomicrograph of liver tissue sections stained by H&E stain representing, (a) control group with normal histological structure; (b–c) IM receiving group showing, (b) diffuse hepatocellular cytoplasmic vacuolization with severe congestion in central vein (arrow), sinusoids (arrowhead), and portal vein (star). (c) Portal triad showing moderate mononuclear congestion (star) and inflammatory cells infiltration (arrow). (d–f) HFM receiving group showing, (d) large focal area of hepatocellular coagulative necrosis (arrow) infiltrated with mononuclear inflammatory cells. (e) Large coalescent area of hepatic hemorrhage (star). (f) Portal triad showing severe congestion (star), edema, and mild inflammatory cells infiltration (arrow)

Liver tissue sections of the control rat showing normal histological structure (Fig. 2a). On the other hand, liver sections of IM receiving group showed moderate to severe histopathological alterations. There were severe diffuse hepatocellular cytoplasmic vacuolization and individual hepatocellular necrosis (Fig. 2b). Extensive congestion in the central vein and hepatic sinusoids were also recorded with lymphocytic infiltration. Portal triad showed congestion, moderate inflammatory cells infiltrations, and fibroplasia (Fig. 2c). Regarding HFM receiving group, liver sections showed severe diffuse hepatocellular cytoplasmic vacuolization and extensive congestion of the central vein, sinusoids, and portal vein. Hepatocellular coagulative necrosis with either zonal centrilobular or random focal distribution was noticed with or without mononuclear inflammatory cells infiltration (Fig. 2d). In addition, focal to coalescent areas of hemorrhage were also observed in some sections (Fig. 2e). Portal triad showed severe inflammatory cells infiltration (Fig. 2f).

**Fig. 3:**
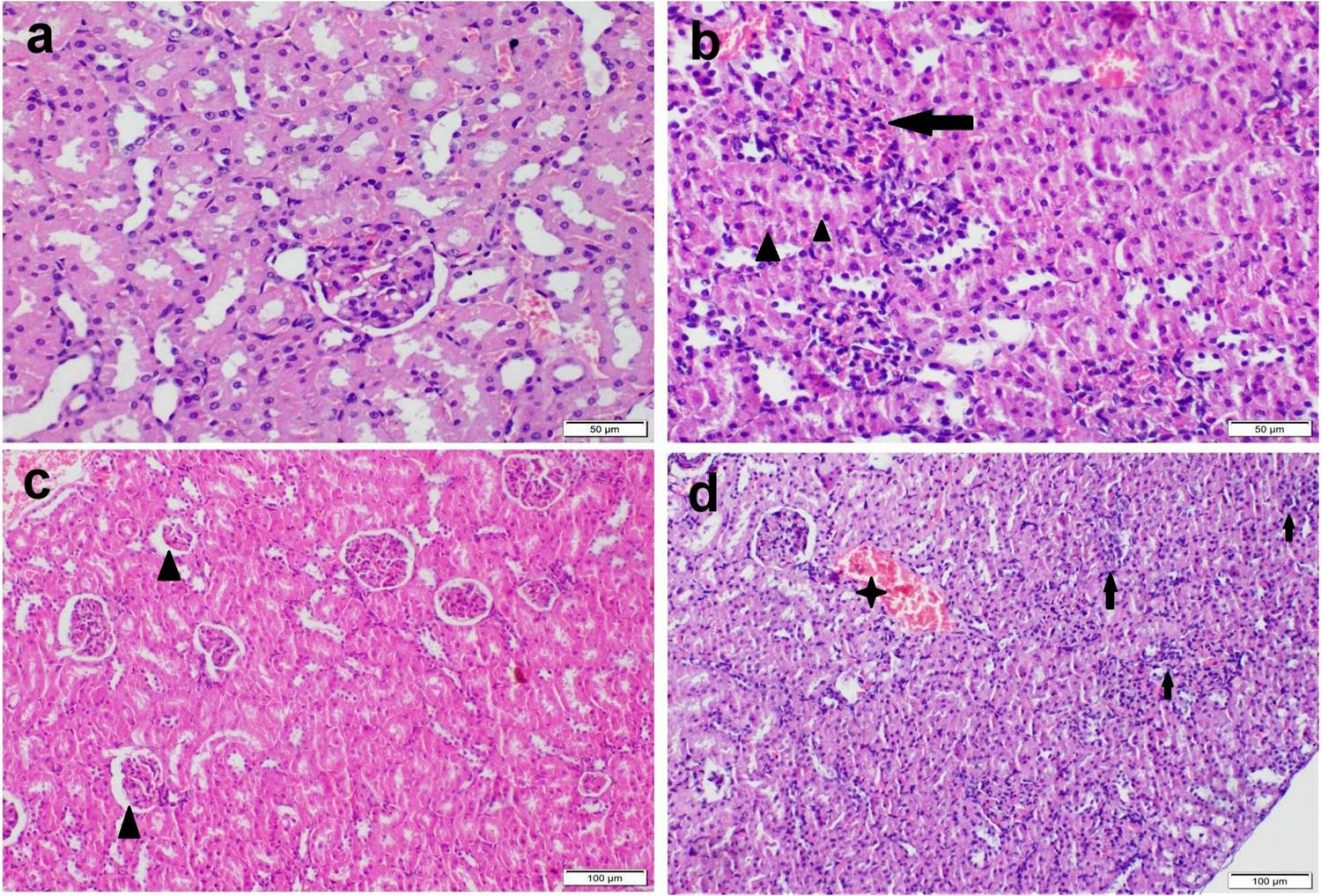
Photomicrograph of kidney tissue sections stained by H&E stain representing, (a) control group with normal histological structure. (b) IM receiving group showing severe congestion in glomerular capillary tuft (arrow), and interstitial bl vs with mild individual cell necrosis in renal tubular epithelium (arrowhead). (c–d) HFM receiving group showing, (c) glomerular atrophy (arrowhead) with widening of Bowman’s capsule. Note: degeneration and necrosis of renal tubular epithelial cells. (d) Severe congestion in glomeruli and renal bl vs (star) with interstitial inflammatory cells infiltration (arrows)

**Fig. 4:**
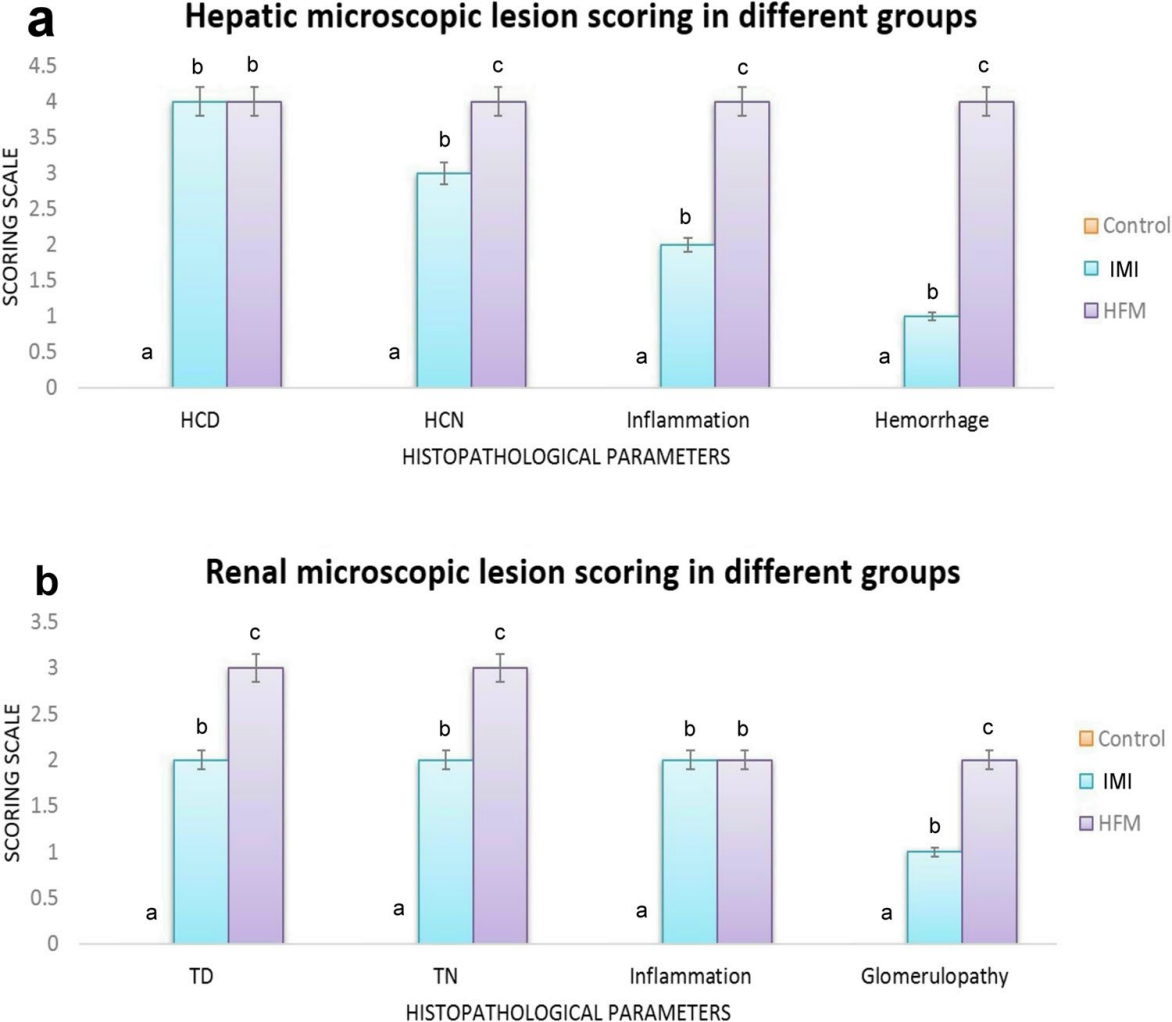
Bar chart representing the microscopic lesion scoring in liver and kidney sections of different groups. Values are presented as median. (n = 5 sections representing 5 rats/group). Values with different letters are significantly different at P ≤ 0.05. Abbreviations: Control (C), Imidacloprid (IM) and hexaflumuron (HFM)

Kidney tissue sections of the control rat showing normal histological structure (Fig. 3a). While those obtained from a rat in IM receiving group showed mild to moderate nephrotoxic nephrosis. Renal tubular epithelial cells showed granular and vacuolar swelling. Most glomeruli showed congestion of the capillary tuft with hypercellularity (Fig. 3b). Interstitial tissue showed severe congestion and mild inflammatory cells infiltration. Concerning HFM receiving group, kidney sections showed severe nephrotoxic nephritis and glomerulopathy. Some glomeruli showed atrophy of glomerular tuft with the widening of bowmen’s capsule while others showing vacuolation, congestion, and/or hypercellularity (Fig. 3c). Renal tubular epithelium showed severe degeneration and necrosis with intraluminal renal cast and droplets. Interstitial tissue showed severe congestion, hemorrhage, and focal inflammatory cells infiltration (Fig. 3d). The result of hepatic and renal lesion scoring was illustrated in Fig. 4 and noticed the highest score in all parameters in the group receiving HFM. Moreover, IM receiving group showed an increase in lesion score in all pathological parameters compared with the control group.

### Immunohistochemical studies

**Fig. 5:**
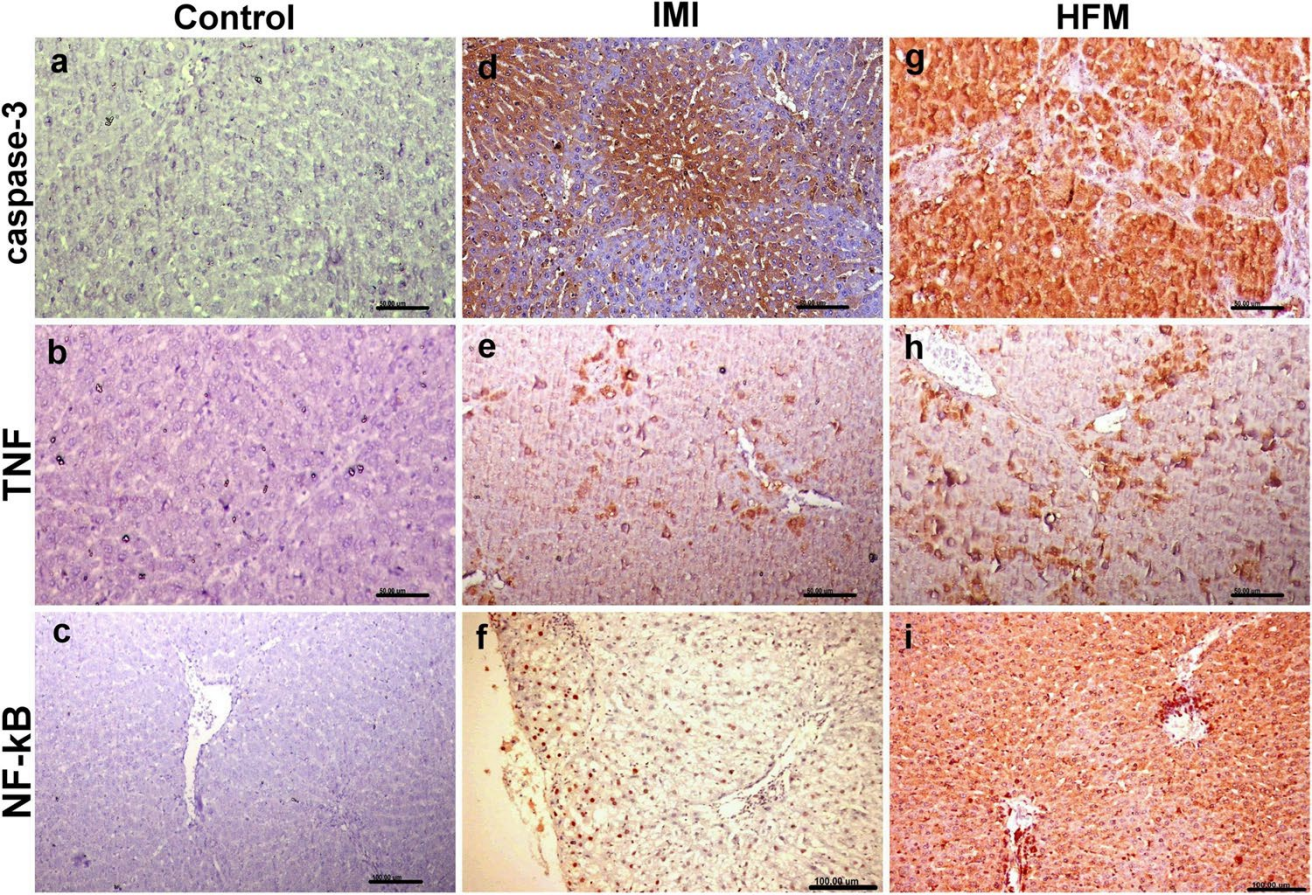
Photomicrographs representing IHC examinations in liver sections of different groups. (a–c) Control group showing normal mild to negative caspase-3, TNF-ὰ, and NF-_K_B expressions respectively. (d–f) Imidacloprid receiving group showing strong to moderate reactions for the above-mentioned immune markers. (g–i) Hexaflumuron receiving group showing strong reactions for all examined immune markers

**Fig. 6:**
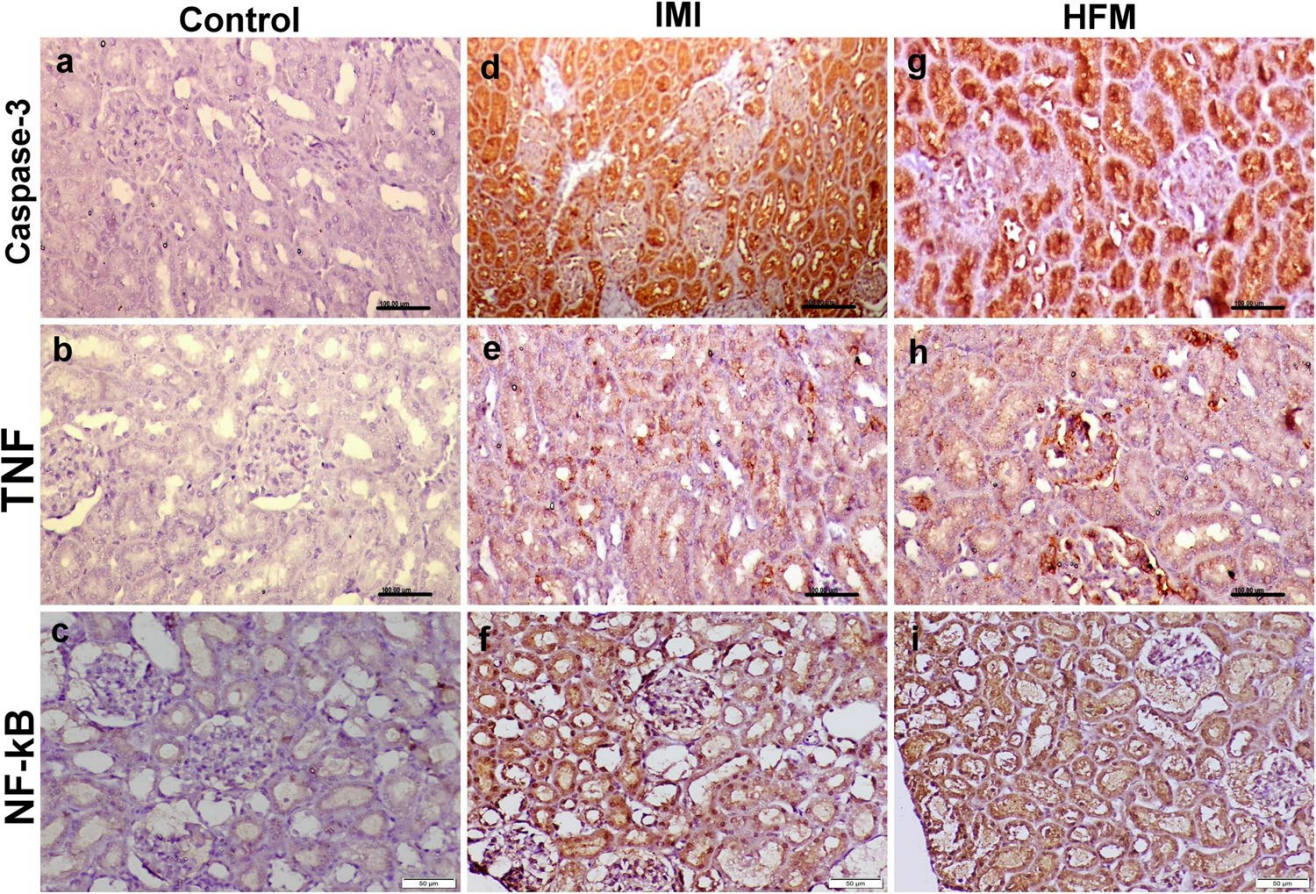
Photomicrographs representing IHC examinations in kidney sections of different groups. (a–c) Control group showing normal caspase-3, TNF-ὰ, and NF-_K_B expressions respectively. (d–f) Imidacloprid receiving group showing strong reactions for the above-mentioned immune markers. (g–i) Hexaflumuron receiving group showing strong reactions for all examined immune markers

**Fig. 7:**
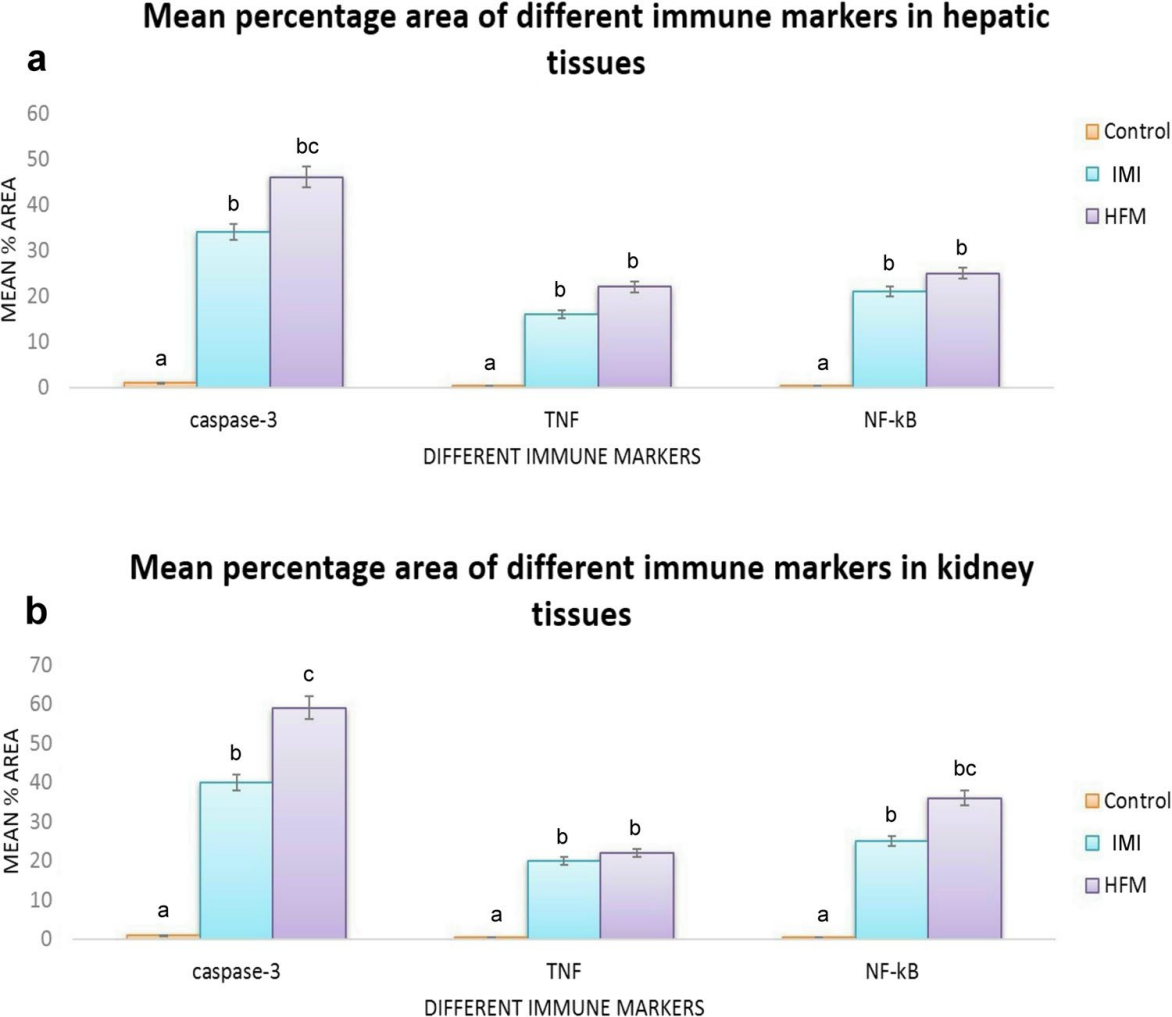
Bar charts representing mean percentage area of caspase-3, TNF-ὰ, NF-_K_B immunopositivity in liver (a), and kidney (b) tissue sections. Values are presented as mean ± SD. (n = 10 low power fields per section, total 5 sections representing 5 rat/group). Values with different letters are significantly different at P ≤ 0.05

There were strong positive expressions of caspase-3, NF-_K_B, and TNF-ὰ in both liver and kidney sections obtained from the group receiving HFM compared with the control group. Moreover, the group receiving IM showed moderate reactions for the above-mentioned immune marker in both liver and kidneys (Figs. 5, 6, 7).

### Quantitative RT-PCR for caspase-3, JNK, HO-1, and Keap-1 genes

**Fig. 8:**
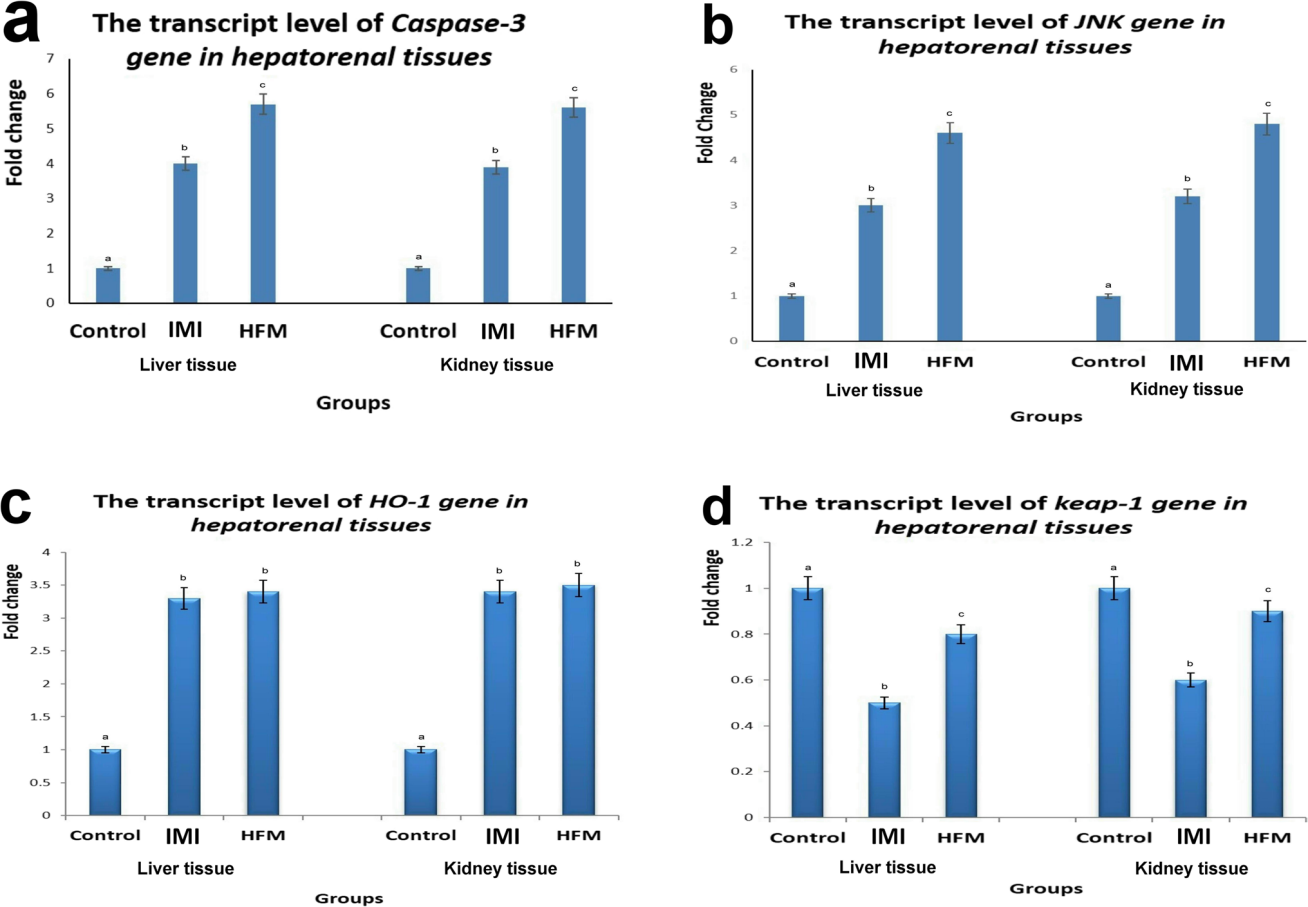
Bar charts representing the transcript levels of caspase-3 (a), JNK (b), HO-1 (c), and Keap-1 (d) genes in liver and kidney tissue homogenates from different groups. Values are presented as mean ± SD. (n = 5 rats/group). Values with different letters are significantly different at P ≤ 0.05. Control (C), Imidacloprid (IM) and hexaflumuron (HFM), c-Jun N-terminal kinases (JNK), Heme oxygenase (HO-1), Kelch Like ECH Associated Protein-1 (Keap-1)

Significant increases in the transcript levels of caspase-3, JNK, and HO-1 genes and a decrease in Keap-1 gene were observed in groups receiving either IM or HFM compared with the control groups. The highest levels of caspase-3, JNK, and HO-1 genes were recorded in the group receiving HFM compared with the IM group. On the other hand, IM group recorded the lowest level of Keap-1 gene when compared with HFM group (Fig. 8).

## Discussion

Exposure to environmental pollution remains a major source of health risk worldwide especially in developing countries (Bolognesi and Morasso 2000). Among the environmental pollution, overexposure to pesticides is considered as one of the causative factors of various health problems in humans and animals (Cheng et al. 2011). The current study is designed to investigate the possible toxic effects of two types of insecticides (IM and HFM) on the liver and kidney of rats through measuring rat’s body weight and behavioral changes, liver and kidney biomarkers, lipid profile, oxidative stress markers, histopathological examination, caspase-3, NF-_K_B, and TNF-ὰ immune markers and measuring mRNA levels of casp-3, JNK, HO-1, and Keap-1 genes to spot the mechanistic way of toxicity of these insecticides in rats.

In the current study, there was a significant decrease in body weight with neurobehavioral alterations of rats after exposure to IM and HFM despite having free access to food suggesting the toxic effect of both pesticides to animal organs that may interfere with the absorption of some nutrients (Ndonwi et al. 2020). In agreement with our findings, several studies confirmed that insecticides caused a decrement in body weight that might be because of the repellent impacts of the insecticide's prompts decrease in food consumption (Werner et al. 2010; Arfat et al. 2014). In addition, severe hepatotoxicity has been reported to induce hepato-encephalopathic neurobehavioral alterations (Hayase et al. 2000).

The results of the present study revealed that IM and HFM induced hepatorenal oxidative stress damage manifested by increased MDA levels and decreased GSH levels triggered by upregulation of HO-1 gene and down-regulation of Keap1 gene. These findings agreed with other reports that showed that lufenuron, belonging to the same hexaflumuron-family, was able to induce oxidative stress damage in the liver of rats (Basal et al. 2020). Also, chronic exposure to IM alters inflammation and oxidative stress markers in the liver and central nervous system of rats (Duzguner and Erdogan 2012). Increasing MDA levels suggest free O_2_ overproduction that initiates DNA damage, protein degradation, lipid peroxidation and tissue damage particularly liver (organ of detoxification) and kidneys (organ of excretion) (Timoumi et al. 2019). HO-1 is a stress-induced isoform located in the endoplasmic reticulum, mitochondria, cell nucleus, and plasma membrane of several cell types mainly the liver and kidneys (Hopper et al. 2018). its upregulation occurred in many conditions associated with oxidative stress, chemical toxicity, and inflammatory reactions (Ferrándiz 2008). Keap1 has been shown to interact with Nrf2, a master regulator of the antioxidant response, which is important for the amelioration of oxidative stress (Wang et al. 2008; Deshmukh et al. 2017). Several studies confirmed that Keap-1 or Nrf2 down-regulation increased mitochondrial ROS production and induced the process of apoptosis and inflammation (Shibata et al. 2008).

The results of oxidative stress evaluations reflected on the histopathological picture of liver and kidneys of rats receiving IM and HFM that showing severe pathological alterations related to ROS overproduction. Several studies reported that ROS overproduction increasing cell and mitochondrial membrane permeability and prompts mitochondrial dysfunction disrupting Na/K/ATPase pump causing ionic imbalance (Khalaf et al. 2020; Morgan et al. 2021) This mechanism explained the observed hepatocellular and renal tubular epithelial cells degeneration in the present study. Furthermore, oxidative stress causing mitochondrial dysfunction and opining mitochondrial transition pores that increase the cytosolic Ca levels which activate several enzymes leading to protein degradation, lipid peroxidation and DNA damage (Mansour and Mossa 2010). Those causing further membrane damage and cell death via necrosis and apoptosis pathway. Apoptosis is a programmed cell death initiated by several factors including ROS production (Kandemir et al. 2017). The key event in apoptosis is the activation of a series of caspase cascades that are segregated into three groups based on their roles in apoptosis (Lakhani 2006). The first group including caspases with a death effector domain as casp-8 and -10 that associated with the cell membrane DRs of TNF-ὰ and Fas and inducing apoptosis via their death domains while, the second group including caspases with a recruiting domain as casp-1, -4, -5, -2, and -9 (Denecker et al. 2008). Whereas, the last group including effector caspases as casp-3, -6, and -7 that activated by the first two groups of caspases (Mandlekar et al. 2000). Caspase-3 has been known as a marker of both physiological and stress-induced apoptosis in mammalian cells and initiates the apoptotic cascade by activating other caspases (Eldutar et al. 2017). It has an important role in the terminal and execution phase of apoptosis via split the variety of substrates implicated in the typical morphological and biochemical characters noticed in apoptosis (Falschlehner et al. 2007).

JNK belongs to the mitogen-activated protein kinase family that plays an important role in the process of oxidative and nitrosative stress-induced apoptosis (Vlahopoulos and Zoumpourlis 2004). It is activated by several factors including ROS produced by several cytotoxic chemicals and proinflammatory cytokines as TNF-ὰ causing apoptosis through a series of intermediate (Oltmanns et al. 2003). These data suggest our results about strong caspase-3 protein expressions in both liver and kidney sections of both insecticides receiving groups together with up-regulation of mRNA levels of different apoptotic markers as caspase-3 and JNK genes. Thus, our results suggest that both insecticides induced hepatorenal apoptosis through casp3-dependent and independent apoptosis through activation of the JNK pathway. Both mechanisms are mediated by oxidative or nitrosative stress induced by both insecticides either directly via their toxic effects or indirectly via activation of cytokines and chemokines; however, the relationship between these two mechanisms remains elusive.

The observable hepatocellular pathological alterations were confirmed by measuring the hepatocellular integrity through the determination of two biochemical markers, serum ALT and AST. Our results showed a significant elevation in both biochemical hepatic markers indicating a defect in the permeability of cell membrane and cellular necrosis (Manfo et al. 2020, Toor et al., 2012). Our results also showed a decline in the total proteins levels in the IM and HFM groups suggesting a reduction in albumin synthesis in response to hepatocellular damage (Chakroun et al. 2017). Data illustrated in the current study also showed a significant elevation in both urea and creatinine concentrations upon exposure to either IM or HFM suggesting their nephrotoxic potential. These findings can be explained by the histopathological observations that showed severe nephrotoxic nephrosis, nephritis, and glomerulopathy in groups receiving either IM or HFM but the most prominent lesions observed in HFM receiving groups compared with IM group.

The data presented in our study demonstrated a significant elevation in serum glucose levels in both IM and HFM administration indicating a disturbance of carbohydrate metabolism as a result of enhancing the liver breakdown of glycogen. Results obtained by Kim et al (2013) support our findings as IM could affect insulin signaling pathways. Additionally, IM and HFM administration led to a significant elevation in total cholesterol, TD and LDL-C concentrations, while HDL-C level was significantly diminished in both interventions in comparison to the control group. Pesticides were reported to induce oxidative stress associated with mitochondrial dysfunction and impairment of glucose and lipid metabolism (Bonvallot et al. 2018). In addition, pesticides exposure alters lipid homeostasis together with the hepatic and adipocytes lipid storage impairment, thus the lipid level in the blood is affected by pesticides that led to disruption of energy balance (He et al. 2020).

Furthermore, the present study showed moderate-to-severe TNF-ὰ and NF-_K_B expressions in both liver and kidney sections of all insecticide receiving groups. It is reported that there were correlations between insecticides exposure and changes in cytokine activity. Omurtag et al. (2008) reported that pesticides administration resulted in hepatoxicity related to pro-inflammatory cytokine expression (TNF-ὰ) which in turn was increased by oxidative stress. Additionally, several insecticides can trigger ROS production leading to oxidative stress and enhanced activation of the NF-_K_B pathway (Yang et al. 2009). In addition, it induces a significant production of TNF-ὰ and NO in macrophages and thus contributes to inflammatory reactions, cytokine imbalance, and immune dysregulation (Dutta et al. 2008). Videla et al. (2004) have proposed that lindane-induced oxidative stress in the liver triggers DNA binding activity of NF-_k_B, with a consequent increase in the expression of NF-_K_B-dependent genes for TNF-ὰ, therein identifying factors that may mediate the hepatotoxic effect of insecticides. In the current study, the observed increase of MDA levels in the liver and kidneys, correlated with the stimulation of pro-inflammatory cytokine expression suggests that both IM and HFM may mediate its toxicity via activation of NF-_K_B signaling pathway in the chronic phase of inflammation. Furthermore, the present study revealed that HFM is more toxic to liver and kidneys of rats than IM in all studied parameters, but both types of insecticides have the same toxic mechanisms but with different degree of severity. Imidacloprid, belonging to neonicotinoid pesticides, has low toxicity in vertebrates due to its weak interaction with nicotinic receptors in vertebrates, but the ingestion of large amounts of this pesticide caused severe poisoning (Phua et al. 2009). This poisoning may be related to the prolonged absorption and/or elimination of high doses of imidacloprid (Mohamed et al. 2009). Recent studies discussed that most insecticides belonging to benzoylphenyl urea are more toxic to aquatic invertebrates than other pesticide families as neonicotinoid, carbamate, and organophosphate, but data about their toxicity on the vertebrates is still unclear (Yu et al. 2014). The present study revealed that hexaflumuron, belonging to benzoylphenyl urea, has a high impact on liver and kidneys of rats via prominent oxidative stress damage that influences mitochondrial dysfunctions and prompts caspase-3 and JNK-dependent apoptosis.

## Conclusion

From the results of the current study, we can conclude that both IM and HFM insecticides exert severe hepatorenal oxidative stress damage. IM and HFM induced oxidative stress in several ways including ROS production, antioxidants exhaustion, HO-1 upregulation, and inactivation of the Keap-1 gene that inactivate the Nrf2 signaling pathway. In addition, both insecticides activate the JNK signaling pathway via ROS overproduction causing apoptosis via several cascade activation including upregulation of caspase-3 gene and protein overexpression. Activation of NF-_K_B signaling pathway is another mechanism for both insecticides-induced hepatorenal toxicity in rats. Both IM and HFM stimulate cytokines production including TNF-ὰ via several ways such as ROS overproduction and JNK activation which activate nuclear translocation of NF-_K_B within hepatic and renal cells initiate the process of inflammatory reactions. Further studies are needed to investigate the effect of lower dosage levels of these pesticides for a prolonged period to evaluate the effect of chronic or long-term administration of lower dosage levels.

## Data Availability

All data are available on request.

## Abbreviations

ABC: Avidin–biotin-peroxidase complex
ALT: Alanine aminotransferase
AST: Aspartate aminotransaminase
Bax: B-cell lymphoma 2-associated protein-X
Bcl-2: B-cell lymphoma 2
Bwt: Body weight
Casp: Caspase
Cyt-C: Cytochrome-C
DNA: Deoxyribonucleic acid
GSH: Reduced glutathione
H&E: Hematoxylin and Eosin
HDL-C: High density lipoproteins cholesterol
HFM: Hexaflumuron
HO-1: Hemoxygenase-1
IL-1: Interleukine-1
IM: Imidacloprid
JNK: C-Jun N-terminal kinase
LDL-C: Low-density lipoproteins cholesterol
MDA: Malondialdehyde
NF-_K_B: Nuclear factor-kappa protein
NO: Nitric oxide
Nrf2: Nuclear factor erythroid 2–related factor 2
ROS: Reactive oxygen species
RT-PCR: Real Time-Polymerase chain reaction
TG: Triglycerides
TNF-ὰ: Tumor necrosis factor alfa
